# Marker-Negative Pheochromocytoma Associated with Inferior Vena Cava Thrombosis

**DOI:** 10.1155/2017/6270436

**Published:** 2017-06-15

**Authors:** S. Poudyal, M. Pradhan, S. Chapagain, B. R. Luitel, P. R. Chalise, U. K. Sharma, P. R. Gyawali

**Affiliations:** Department of Urology and Renal Transplant Surgery, Tribhuvan University Teaching Hospital, Kathmandu, Nepal

## Abstract

Pheochromocytoma associated with inferior vena cava (IVC) thrombosis is very rare. A 27-year-old female presented with right flank pain and hypertensive urgency. Contrast-enhanced CT abdomen and gadolinium-contrast MRI abdomen revealed right adrenal mass suspicious of malignancy with invasion and compression to the right IVC wall along with IVC thrombus extending from the level of renal veins to the level of confluence with hepatic veins. Her routine laboratory investigations including 24-hour urine fractionated metanephrines, vanillylmandelic acid, and cortisol were normal. Right adrenalectomy with IVC thrombectomy was done. Perioperative period was uneventful. Histopathology of the mass turned out to be pheochromocytoma with thrombus revealing fibroadipose tissue with fibrin. Pheochromocytoma may present with IVC thrombus as well as normal serum and urinary markers. Thus, clinical suspicion is imperative in perioperative management of adrenal mass.

## 1. Introduction

Pheochromocytoma is a rare tumor of the catecholamine-producing cells of the adrenal medulla. Prevalence of the disease may vary but approximately 1 to 2 per 100,000 individuals are diagnosed annually [1]. The classic hallmark of the disease is the triad of headache, episodic sudden perspiration, and tachycardia [1]. The disease is commonly diagnosed biochemically by plasma-free metanephrines or 24-hour urine fractionated metanephrines and localized by imaging modalities like contrast-enhanced CT, MRI, or metaiodobenzylguanidine (MIBG) scintigraphy [2–6]. The sensitivity and specificity of biochemical markers are still 95-96% and 86–91%, respectively [7]. It is likely that biochemical marker-negative pheochromocytoma may be encountered in clinical practice, though large adrenal mass with negative serum marker and inferior vena cava (IVC) thrombus is usually an adrenocortical carcinoma [8, 9]. Pheochromocytoma coexisting with IVC thrombus is very rare [10]. Hence, we report a marker-negative pheochromocytoma which was associated with IVC thrombosis.

## 2. Case Report

**Figure 1:**
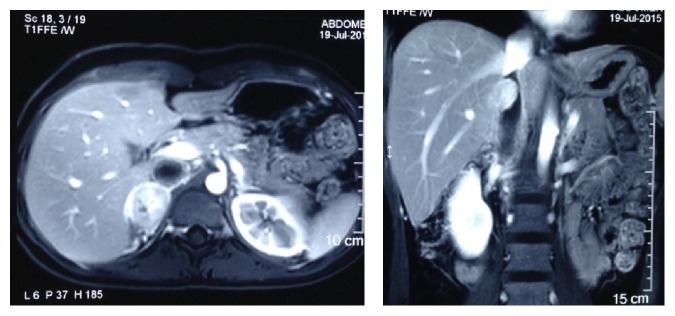
Gadolinium enhanced MRI abdomen axial and coronal view showing right adrenal mass and IVC thrombosis.

**Figure 2:**
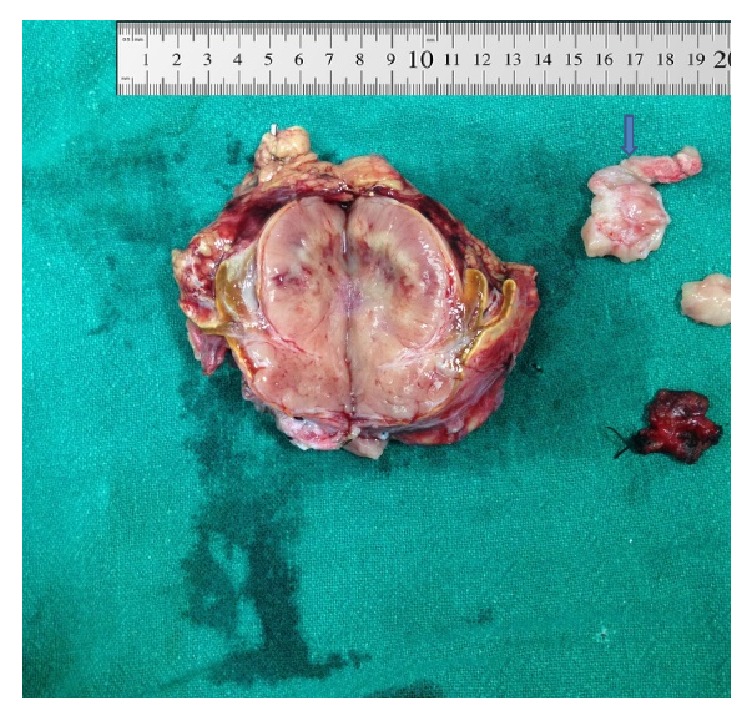
Pheochromocytoma in cut section with IVC thrombus (shown by arrow).

**Figure 3:**
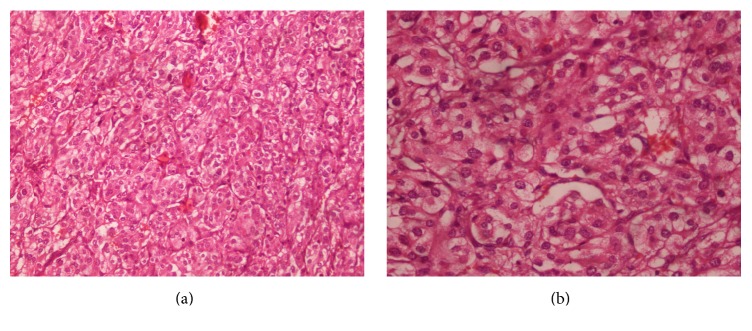
Biopsy showing oval to polygonal tumor cells with abundant granular eosinophilic to clear cytoplasm and oval nucleus. The nests of tumor cells (zellballen pattern) are separated by sustentacular cells ((a) ×200; (b) ×400).

A 27-year-old female presented with intermittent dull-aching right flank pain for one month. It was associated with frontal headache, sweating, and palpitation. She had history of normal vaginal delivery 45 days back. She had no significant past medical and familial history. Blood pressure at presentation was 180/110 mmHg with pulse of 90 beats/min. There was mild tenderness in the right flank with no other significant findings. USG abdomen revealed right adrenal mass. CECT abdomen and gadolinium-contrast MRI abdomen revealed 6 × 4 cm heterogeneously enhancing mass with delayed washout suggestive of right adrenal malignancy with invasion to right IVC wall leading to narrowing and presence of nonenhancing IVC thrombus extending from the level of renal veins to the level of confluence with hepatic veins (Figure 1). Venous Doppler of both lower limbs and echocardiography were unremarkable. Her routine laboratory investigations including serum creatinine, electrolytes, liver function tests, adrenocorticotropic hormone, and cortisol level were normal. Similarly, 24-hour urine fractionated metanephrines, vanillylmandelic acid, cortisol, serum metanephrines, and catecholamines were also within normal limits. With a provisional diagnosis of right adrenocortical carcinoma with IVC thrombosis, right adrenalectomy with IVC thrombectomy was planned. Two weeks prior to surgery, prazosin was started and titrated to 5 mg orally per day to control her blood pressure. Subsequently, 50 mg atenolol was added orally once a day for tachycardia. Lower molecular weight heparin was also started. With right anterior subcoastal approach, right adrenal mass was mobilized. Control of right renal artery and vein, left renal vein, and infrarenal IVC was taken. Right lobe of the liver was mobilized medially and infradiaphragmatic control of the IVC was attempted but failed. Therefore, with right thoracotomy, supradiaphragmatic control of IVC was taken. Serial clamping of infrarenal IVC, right renal artery and vein, left renal vein, and supradiaphragmatic IVC was undertaken. Pringle maneuver was applied to control bleeding from the hepatic veins. Longitudinal venotomy extending from the right adrenal vein to IVC was given and thrombus was removed along with the adrenal mass. The intraoperative finding revealed 6 × 5 cm encapsulated firm adrenal mass not invading surrounding organs and organized firm greyish thrombus mixed with clots extending to IVC up to the level just caudal to confluence of hepatic vein. On cut section, the mass was flesh coloured with central pale area containing some hemorrhagic areas (Figure 2). Intraoperative and postoperative period were uneventful with total of three units of packed red blood cell transfusion. Postoperatively, her blood pressure was normal without antihypertensive drugs and she was discharged on seventh postoperative day on aspirin. Histopathology of the mass turned out to be pheochromocytoma with thrombus revealing fibroadipose tissue with fibrin (Figure 3). There was absence of mitotic figures, necrosis, and capsular and vascular invasion. Immunohistochemically, the tumor cells were positive for chromogranin A. MIBG scan done after 6 weeks and CECT abdomen done after 6 months were unremarkable.

## 3. Discussion

Pheochromocytoma coexisting with IVC thrombus is very rare. All IVC thrombus associated with adrenal mass is not tumor thrombus [11]. So, there can be bland thrombus in IVC as a result of hypercoagulable status of the patient, stasis of blood due to obstruction, and trauma to the vessel as described by Virchow [12]. Kota et al. reported a single case of left pheochromocytoma with IVC thrombus in India. In his case report, the case underwent laparoscopic adrenalectomy and IVC thrombus was managed as bland thrombus with anticoagulant postoperatively [10]. In our case, the patient was postpartum which is a known cause for hypercoagulability [13, 14]. Similarly, the gradual compression of IVC by adrenal tumor resulted in stasis of blood and IVC thrombosis subsequently.

Though both CECT abdomen and gadolinium-contrast MRI were suggestive of IVC wall invasion by adrenal tumor, the IVC thrombus was nonenhancing in both, which was indicative of bland thrombus [15, 16]. Guo et al. studied 25 cases of renal cell carcinoma with IVC thrombus and reported that multidetector computed tomography and magnetic resonance imaging are comparable and more effective than abdominal ultrasound in diagnosing inferior vena cava tumor thrombus in renal cell carcinoma but none of the three methods can detect inferior vena cava wall invasion [15]. Similarly, there was no IVC wall invasion intraoperatively in our case. There are few case reports of malignant adrenal and extra-adrenal pheochromocytoma associated with IVC thrombosis [17, 18].

Plasma metanephrines or 24-hour urine fractionated metanephrines, being highly sensitive, are the gold standard for diagnosing pheochromocytoma. In our case, it was very difficult to label the adrenal tumor as pheochromocytoma as it was associated with IVC thrombus and 24-hour urinary metanephrines were not raised. In spite of that, with typical history and radiological finding, the case underwent surgery with catecholamine blockade as that of pheochromocytoma. Hence, the hazardous complication of hypertensive crisis was avoided during the surgery. Diagnosing pheochromocytoma can be sometimes very challenging in case of negative markers [7, 19]. Heavner et al. reported nine percent of pheochromocytoma to be marker-negative in their series. Thus, they advocated that patients with adrenal masses with presentation suggesting catecholamine excess with normal labs may warrant a metaiodobenzylguanidine scan or repeat testing to avoid missing pheochromocytoma [19]. Though MIBG scan can be used to diagnose pheochromocytoma in case of high clinical suspicion with high sensitivity (83% to 100%) and superb specificity (95% to 100%), it is still falsely negative in some group of patients [6, 20].

The most difficult task in case of marker-negative pheochromocytoma is ruling out metastatic disease and following up the case for surveillance which is recommended lifelong [21–23]. Malignant pheochromocytoma is defined as pheochromocytoma associated with clinical metastasis [24]. Metastatic disease is routinely ruled out by assessing plasma or urine metanephrines two weeks after adrenalectomy [23]. MIBG scan is done in cases with negative markers even though it is not 100% sensitive. In our case, MIBG done was negative for residual and metastatic disease. Recent studies show that 18F-FDG PET exhibited better accuracy than 123I-MIBG in nearly all patients, especially for identification of metastatic disease [25–27].

Genetic testing of the RET, VHL, SDHB, and SDHD gene mutations is advocated in patients younger than fifty years [23]. As complete immunohistochemistry and genetic testing services are not available in the country, these tests could not be done.

## 4. Conclusion

Pheochromocytoma may coexist with IVC thrombus. In addition, it may present with normal serum and urinary markers. Thus, clinical suspicion is of paramount importance in perioperative management of adrenal mass.
